# Examination of thromboxane synthase as a prognostic factor and therapeutic target in non-small cell lung cancer

**DOI:** 10.1186/1476-4598-10-25

**Published:** 2011-03-09

**Authors:** Mary-Clare Cathcart, Kathy Gately, Robert Cummins, Elaine Kay, Kenneth J O'Byrne, Graham P Pidgeon

**Affiliations:** 1 Department of Surgery, Institute of Molecular Medicine, Trinity Health Sciences Centre, St. James's Hospital, Dublin 8, Ireland; 2 Department of Clinical Medicine, Institute of Molecular Medicine, Trinity Health Sciences Centre, St. James's Hospital, Dublin 8, Ireland; 3 Department of Pathology, Beaumont Hospital, Dublin 9, Ireland

## Abstract

**Background:**

Thromboxane synthase (TXS) metabolises prostaglandin H2 into thromboxanes, which are biologically active on cancer cells. TXS over-expression has been reported in a range of cancers, and associated with a poor prognosis. TXS inhibition induces cell death *in-vitro*, providing a rationale for therapeutic intervention. We aimed to determine the expression profile of TXS in NSCLC and if it is prognostic and/or a survival factor in the disease.

**Methods:**

TXS expression was examined in human NSCLC and matched controls by western analysis and IHC. TXS metabolite (TXB_2_) levels were measured by EIA. A 204-patient NSCLC TMA was stained for COX-2 and downstream TXS expression. TXS tissue expression was correlated with clinical parameters, including overall survival. Cell proliferation/survival and invasion was examined in NSCLC cells following both selective TXS inhibition and stable TXS over-expression.

**Results:**

TXS was over-expressed in human NSCLC samples, relative to matched normal controls. TXS and TXB_2 _levels were increased in protein (*p *< 0.05) and plasma (*p *< 0.01) NSCLC samples respectively. TXS tissue expression was higher in adenocarcinoma (*p *< 0.001) and female patients (*p *< 0.05). No significant correlation with patient survival was observed. Selective TXS inhibition significantly reduced tumour cell growth and increased apoptosis, while TXS over-expression stimulated cell proliferation and invasiveness, and was protective against apoptosis.

**Conclusion:**

TXS is over-expressed in NSCLC, particularly in the adenocarcinoma subtype. Inhibition of this enzyme inhibits proliferation and induces apoptosis. Targeting thromboxane synthase alone, or in combination with conventional chemotherapy is a potential therapeutic strategy for NSCLC.

## Introduction

Lung cancer is the leading cause of cancer related death in the developed world, accounting for 12% of deaths worldwide [1]. Median survival for the majority of patients with advanced non-small cell lung cancer (NSCLC) is 18 months and 9 months for locally advanced or metastatic disease respectively [2]. Current therapeutic strategies are relatively ineffective, which is reflected by an overall survival rate of just 15% [3].

Arachidonic acid (AA) can be converted to various eicosanoids by enzymes such as cyclooxygenase (COX), lipoxygenase (LOX), or epoxygenases (cytochrome P-450). The cyclooxygenase enzymes consist of two isoforms, COX-1 and COX-2, which catalyze the first step in the generation of downstream prostanoids from arachidonic acid [4]. COX-derived prostanoids are involved in a wide range of physiological processes, but have also been implicated in a range of disease states, such as arthritis, heart disease, and pulmonary hypertension [5]. In the past number of years, there has been significant interest in the role of COX-2 in cancer development and progression. Expression of this enzyme has been associated with a poor prognosis in lung cancer [6-8], while a potential role for COX-2 in lung cancer chemoprevention has been investigated in a number of clinical trials [9-11]. However, chronic administration of high concentrations of selective COX-2 inhibitors has been associated with an increased risk of adverse cardiovascular events [12-14]. Recent studies suggest that the tumour-promoting effects of COX-2 over-expression may be attributable to downstream products of AA metabolism. Increased COX-2 expression is associated with increased levels of downstream enzymes required for prostanoid synthesis, such as prostaglandin E_2 _synthase (PGE-S), prostaglandin D_2 _synthase (PGD-S), and thromboxane A_2 _synthase [15]. However, the relationship of prostanoid profile to cancer growth is not fully understood.

Thromboxane synthase (TXS) activity was first described in platelets [16]. The enzyme was later purified as a 60 kDa hemoprotein with spectroscopic characteristics of the cytochrome P-450 family [17]. TXS metabolises the cyclooxygenase product, prostaglandin H2, into thromboxanes, which are biologically active on cancer cells. TXA_2 _is a potent vascoconstrictor and bronchoconstrictor, as well as a potent promoter of platelet aggregation [18,19]. TXS and its product, TXA_2_, have been shown to promote proliferation, invasion, metastasis and angiogenesis in a variety of cancers [20-24]. TXS over-expression has been reported in thyroid, prostate, colorectal, and bladder cancer [20-22,24,25]. Over-expression of this enzyme has been associated with a significant reduction in survival in bladder cancer [21]. In addition, analysis of prostate tumour specimens revealed increased TXS levels in patient samples of advanced stage and grade [22,25]. Thromboxane synthase expression has been associated with tumour growth in a variety of cancers, both *in-vivo *and *in-vitro *[21,24,26,27]. A number of these studies have demonstrated a significant reduction in tumour cell growth following selective TXS inhibition [21,24,26], suggesting that targeting this enzyme may have therapeutic efficacy in cancer. Specific thromboxane synthase inhibition has been shown to induce apoptosis, providing a further rationale for therapeutic intervention [26,28].

While the expression and role of thromboxane synthase has been examined in a number of cancers and has demonstrated significant promise, this enzyme has not yet been investigated in NSCLC. Due to the clear role of COX-2 in NSCLC pathogenesis, investigation of the downstream TXS enzyme would be of significant interest and relevance. The aim of this study was therefore to examine the expression profile of thromboxane synthase in NSCLC, relative to matched controls. We aimed to correlate TXS expression patterns with a range of clinical parameters, including overall survival (to determine if it is prognostic in the disease). Finally, we also wished to examine the effect of both TXS inhibition and TXS overexpression on *in-vitro *NSCLC survival mechanisms.

## Methods

### Patients and tumour specimens

Patients who had received primary chemotherapy (neoadjuvant) treatment were excluded from this study. For protein analysis, a series of 26 fresh-frozen NSCLC specimens (13 Adenocarcinomas and 13 Squamous Cell Carcinomas) with corresponding matched normal tissue from the same individual were collected at surgery at St. James's Hospital. All samples were evaluated by a pathologist immediately following dissection.

For generation of a NSCLC tissue microarray (TMA), paraffin blocks from 204 patients who underwent chemotherapy with surgical resection at St. James's Hospital (2000-2005) for NSCLC of varying histology, stage, and grade were collected. Patients who underwent pre-operative chemotherapy, or were found to have stage IV disease were excluded. Patient tumours were staged according to the tumour-node-metastasis (TNM) classification and histologically classified according to the World Health Organization guidelines. The NSCLC tissue samples were classified as 1) adenocarcinoma, 2) squamous cell carcinoma, and 3) others. Patients range in age from 41 years to 86 years. Patients were followed up by the tumour registries for survival time and outcome with median follow-up of 60 months (ranging from 1 to 88 months).

### Generation of NSCLC tissue microarrays

Tissue microarrays were assembled using a tissue arraying instrument (Beecher Instruments, Silver Spring, MD). A 3 mm diameter stylet was used for sampling, with three replicate core samples of tumour taken from different regions in each donor block to account for tumour heterogeneity. Four μM sections of the resulting microarray blocks were cut on a microtome (Leica Microsystems Inc., IL, USA) and dried onto poly-L-lysine-coated glass slides (BDH Laboratory Supplies, Poole, England).

### RNA isolation and reverse-transcription PCR analysis

A-549 and SKMES-1 cells for RNA analysis were collected in 1 mL of Tri-Reagent (Molecular Research Center, OH, USA). Total RNA was subsequently isolated according to manufacturers' instructions. RNA quantification was determined spectrophotometrically (Nanodrop technologies, DE, USA), with RNA concentration subsequently used to carry out cDNA synthesis (reverse-transcription). First strand cDNA was prepared using Superscript III according to the manufacturers' instructions (Invitrogen Corporation, CA, USA). cDNA was generated using random primers (Promega, WI, USA), and synthesis reactions were carried out for 3 h at the appropriate temperature. Expression of TXS and *β*-actin was examined by RT-PCR using previously published primers [20,29].

PCR cycling conditions were as follows:

*β*-actin: 95°C for 5 min, followed by 35 cycles of (1 min at 94°C, 1 min at 56°C, 1 min at 72°C), with a final extension at 72°C for 10 min.

TXS: 94°C for 5 min, followed by 35 cycles of (30 s at 94°C, 30 s at 55°C, 45 s at 72°C) with a final extension at 72°C for 10 min.

*β*-actin levels were utilized as the internal loading control for each sample analysed.

### Western blot analysis

Primary tissue protein lysates were extracted from NSCLC specimens and matched normal lung tissue using Tri-Reagent (MRC), according to the manufacturers' instructions. Protein samples (25 μg) were separated by pre-cast SDS-PAGE and transferred to PVDF membranes. Membranes were probed overnight at 4°C with a primary antibody directed against thromboxane synthase (1:250 dilution, Cayman, Michigan, USA). Following probing, blots were stripped and re-probed for *β*-actin (1:20,000, Merck Biosciences, Germany) to normalize for loading differences. Densitometric analysis was carried out on all TXS tumour/normal matched western blots and corresponding *β*-actin controls. Analysis was carried out using TINA (version 4.0) software (Raytest, Germany). Protein expression was normalized to *β*-actin controls, and was expressed as a ratio of TXS expression: *β*-actin expression.

### Immunohistochemical staining

COX-2 and TXS staining of NSCLC tissue was carried out manually using Vectastain Elite Kits (Vector labs, Burlingame, CA, USA). Immunostaining was carried out using a rabbit polyclonal IgG specific for COX-2 (1:600 dilution, Cayman Chemical), or a rabbit polyclonal IgG specific for TXS (1:500 dilution, Cayman Chemical). Slides were incubated in the primary antibody for 1 h at room temperature (COX-2), or overnight at 4°C (TXS). Staining was visualised using a ScanScope GL digital slide scanner and Aperios ImageScope software (Aperio Technologies Inc., CA, USA).

### Quantification of staining intensity

Staining in NSCLC tissue micro-array tissue was blindly scored and quantified by three independent observers (M.C.C., K.G. and G.P.). Expression levels within tumour tissue was quantified across three representative cores per patient as a product of the staining intensity (0 = negative, 1 = weak, 2 = moderate, 3 = strong) x percentage cells stained (< 25%, < 50%, < 75%, < 100%). Staining intensity was quantified under high magnification (x 20), using Aperio Image Scope software (Aperio Technologies). Expression patterns were correlated with a range of clinical parameters such as patient demographics (gender), tumour classifications (tumour subtype, stage, grade, and nodal status) and overall survival.

### Enzyme immunoassay analysis of metabolite generation

Plasma levels of TXB_2 _(thromboxane A_2 _metabolite) were measured in a retrospective panel of NSCLC plasma samples and age-matched controls (50 chemo-naive patient samples, 19 cancer-free controls). Control samples were taken from patients with no known history of cancer. Plasma samples were isolated from whole blood by centrifugation at 3,000 × *g *for 10 min. Enzyme immunoassay kits (Assay Designs, MI, USA) were used to determine TXB_2 _levels in plasma isolated from NSCLC patients and age-matched controls. The assay was carried out according to manufacturers' instructions.

### *In-vitro *experiments

#### Cell culture and chemicals

Two lung cancer cell lines, A-549 (adenocarcinoma) and SKMES-1 (squamous cell carcinoma) were obtained from and the American Type Culture Collection (Rockville, MD, USA). They were maintained in a humidified atmosphere of 5% CO2 in air at 37°C. A-549 cells were maintained in F-12 (Hams) medium, supplemented with 10% (v/v) foetal bovine serum (FBS), penicillin streptomycin, and L-glutamine. SKMES-1 cells were maintained in Modified Eagle's Medium + Earle's medium (MEM), supplemented with 10% (v/v) FBS, penicillin streptomycin, L-glutamine and non-essential amino acids. Subculturing was carried out when the cells reached 80% confluency. The selective thromboxane synthase inhibitor, ozagrel, was obtained from Cayman.

### Generation and characterisation of stable transfectant clones

Human TXAS cDNA was a gift from Prof. Kenneth V. Honn (Wayne State University, Detroit, MA, USA). A 1.5 Kb DNA fragment containing the entire TXAS coding sequence was digested from pBluescript plasmid DNA and inserted into the site of the mammalian vector pcDNA3.1(-) (Invitrogen Corporation, CA, USA) to generate pcDNA3.1(-)-TXAS. The two mammalian vector constructs were individually transfected into SKMES-1 cells. Stable transfection of vector constructs were carried out using FuGENE 6™ transfection reagent (Roche Diagnostics Ltd., Sussex, UK). Approximately 3 × 10^5 ^cells were cultured in their respective supplement-free medium. Cells were then transfected with either 1 μg pcDNA-TXS/3.1(-), or pcDNA-3.1(-) (control vector) in antibiotic-free media, containing 3 μL/mL FuGENE 6™, according to manufacturers' instructions. Following transfection, cells were further incubated for 24 h at 37°C. Antibiotic selection was then carried out by treating the cells with Geneticin G-418 (800 μg/mL). Following seven days of antibiotic selection, single cell colonies were selected and amplified. RNA and protein was the isolated from amplified clones isolated in order to examine relative TXS expression levels. The TXS clone with the highest TXS expression level was chosen for further analysis (along with corresponding empty vector control cells).

### Cell proliferation analysis

A-549 and SKMES-1 lung cancer cells were seeded at a concentration of 5 × 10^3^/well into 96-well plates and allowed to adhere at 37°C overnight. Following overnight incubation in serum-depleted media, cells were treated for 24 h with and without various concentrations (50 nM, 500 nM, 5 μM) of a selective thromboxane synthase antagonist (Cayman). Cell proliferation was then assessed by BrdU cell proliferation assay, according to the manufacturers' instructions (Roche).

### Annexin-V-FITC/Propidium Iodide Apoptosis Assay

Cells were seeded in 6-well plates (at a dilution of 2.5 × 10^4^, or 5 × 10^4 ^cells/well), which were allowed to adhere at 37°C overnight. Following this, cells were incubated for 48 h or 72 h in serum-depleted media (0.5% FBS). Cells and supernatants were then collected and transferred into flow tubes (BD Biosciences, MA, USA) for annexin-V-FITC apoptosis analysis by flow cytometry (FACS). Cells were prepared for FACS analysis according to manufacturers' instructions (Roche). Apoptosis levels were measured using a FACS Caliber flow cytometer (BD Biosciences), with data analysis carried out using dot plot analysis.

### High content screening analysis

The effects of selective thromboxane synthase inhibition on apoptosis in A-549 and SKMES-1 cell lines was also examined by High Content Screening using the GE Analyser 1000 (GE/Amersham Biosciences, NJ, USA). Briefly, A-549 and SKMES-1 lung cancer cells were set-up in 96-well plates, and treated as previously described. Following treatment, a Multi-Parameter Apoptosis HitKit was used to assess three fundamental parameters related to apoptosis (nuclear morphology, mitochondrial mass/membrane potential and changes in *f*-actin content), according to manufacturers' instructions (Cellomics Inc., PA, USA). Stained plates were sealed and run on a GE Analyser 1000.

### Cell death detection ELISA and DNA laddering

A-549 and SKMES-1 lung cancer cells were seeded at a concentration of 5 × 10^4^/well into 12-well plates and allowed to adhere at 37°C overnight. Following overnight incubation in serum-depleted media, cells were treated for 24 h with and without various concentrations of the selective TXS antagonist, ozagrel (Cayman). The generation of cytoplasmic histone-associated DNA fragments (mono- and oligo nucleosomes) following apoptosis was then measured by Cell Death Detection ELISA (Roche). Sample preparation and the ELISA procedure were both carried out according to manufacturers' instructions. A similar experimental setup was carried out in 75 cm^2 ^flasks, and apoptosis confirmed by DNA laddering analysis using a DNA-laddering kit (Roche).

### Cell invasion assay

Tumour cell invasion was examined using a 96-well Cell Invasion Assay (Chemicon International, CA, USA). Briefly, 1 × 10^5 ^cells in 100 μL serum-free media were added per well to an invasion chamber, which had previously been rehydrated with serum-free media. The 96-well plate was then covered and incubated for 24 h at 37°C. Invasion analysis was then carried out according to manufacturers' instructions.

### Statistical analysis

Statistical comparison of expression levels of these enzymes between tumour and matched control protein, and plasma samples was calculated using a 2-tailed Students *t*-test. Correlation analysis was carried out using a Linear (Pearson) Correlation test. Association between clinicopathological features and expression of these enzymes was assessed using a 2-tailed Students *t*-test for continuous data. Overall survival was examined using Kaplan-Meier's survival curves, with differences in survival assessed by the log-rank test. Statistical analysis for *in-vitro *experiments was carried out using ANOVA, with post-hoc analysis carried out using Tukey-Kramer multiple comparisons test. Results are expressed as mean ± SEM, with data taken as significant where *p *< 0.05. All statistical analysis was carried out using InStat Statistical Software Package version 3.0 with survival analysis carried out using SPSS version 16.0 (SPSS Inc., Chicago, IL, USA).

## Results

### Thromboxane synthase expression in retrospective NSCLC protein samples, relative to matched normal controls

**Figure 1:**
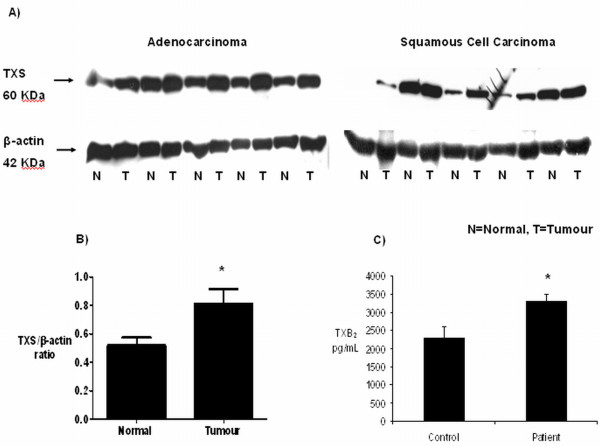
**Expression of TXS and its metabolite TXB_2_ in tumour and matched control patient samples.** A) Expression of TXS in a retrospective panel of tumour/normal matched protein samples. Over-expression of TXS protein was observed in adenocarcinoma and squamous cell carcinoma samples, relative to matched normal (n = 13/group). B) All blots were stripped and re-probed for β-actin to normalise for loading differences (N = normal, T = matched tumour). C) Thromboxane metabolite generation in NSCLC plasma samples and age-matched controls. Plasma levels of the thromboxane metabolite, thromboxane B_2_, were significantly higher in plasma from NSCLC patients than in age-matched controls (C; * *p <*0.01; n = 49 patient, 19 controls).

Examination of TXS expression in a panel of NSCLC protein samples revealed an over-expression of TXS in tumour samples, relative to matched normal controls (Figure 1A). Densitometric analysis confirmed these observations, with TXS expression significantly (*p *< 0.05) higher in tumour samples, relative to matched normal controls (0.81 ± 0.1 tumour *vs*. 0.52 ± 0.06 normal, Figure 1B). When broken into tumour subtype, increased TXS expression was observed in 77% of adenocarcinoma (0.74 ± 0.1 tumour *vs*. 0.54 ± 0.08 normal, not significant, *p *= 0.08) and 77% of squamous cell carcinoma (0.88 ± 0.19 tumour *vs*. 0.5 ± 0.1 normal, *p <*0.05) samples screened (data not shown). These results indicate that thromboxane synthase expression is increased in a significant proportion of patients with NSCLC.

### Thromboxane A_2 _metabolite levels in NSCLC plasma samples and age-matched controls

As thromboxane synthase was increased in our retrospective panel of tumour/normal matched NSCLC protein samples, we wished to determine if this increase in expression at the protein level would be reflected in the downstream generation of the TXA_2 _metabolite, TXB_2_. TXB_2 _levels were measured in a panel of NSCLC plasma samples and age-matched cancer-free controls by enzyme immunoassay (EIA). Measurement of TXB_2 _levels in human plasma would be indicative of circulating levels in the blood. Thromboxane B_2 _levels were found to be significantly (*p <*0.01) higher in plasma samples taken from patients with NSCLC, relative to age-matched controls (3307 ± 189 pg/mL versus 2207 ± 317 pg/mL; n = 49 NSCLC patient, 19 cancer-free controls; Figure 1C), supporting previous observations at the protein level.

### Examination of the thromboxane synthase expression pattern in NSCLC tissue samples and matched normal controls

**Figure 2:**
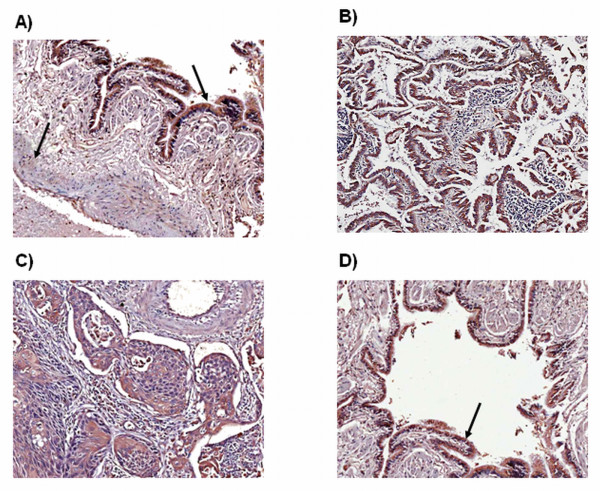
**Expression of PGIS and TXS in a retrospective panel of tumour/normal matched tissue samples**. TXS was weakly expressed in the smooth muscle of normal pulmonary vessels, with a moderate expression observed in pulmonary epithelial cells (A). In tumour sections, TXS expression was observed to a varying degree in both adenocarcinoma (B) and squamous cell carcinoma (C) tissue. A weak TXS expression was observed in vascular smooth muscle cells of the tumour vasculature (C), with strong expression observed in tumour epithelial cells (D). Magnification ×10.

A panel of retrospective lung tumour (adenocarcinoma and squamous cell carcinoma) and matched normal tissue sections were stained by IHC for thromboxane synthase to determine localisation of tissue expression (n = 10 adenocarcinoma and 10 squamous cell carcinoma). TXS was found to be weakly expressed in vascular smooth muscle cells and moderately expressed in pulmonary epithelial cells of normal tissue (Figure 2A). In matched tumour sections, TXS was found to be expressed to a varying degree in both adenocarcinoma (Figure 2B) and squamous cell carcinoma tissue (Figure 2C). In the tumour supplying vessels, TXS expression was strong in bronchial epithelial cells (Figure 2D).

### Tissue microarray analysis of COX-2 and thromboxane synthase in NSCLC: correlation with clinical parameters

A 204-patient NSCLC tissue microarray generated using patients presenting to St. James's Hospital, Dublin, was stained for both COX-2 and TXS expression. COX-2 was expressed to a varying degree in NSCLC tumour tissue. A large number (approximately 30%) of patient samples were found to be negative for COX-2 expression. However, COX-2 expression was detected in a majority of patient samples, where it was found to be highly localised to specific areas of the tumour tissue. Quantification of COX-2 expression levels revealed COX-2 expression was significantly (*p <*0.05) higher in adenocarcinoma tissue, relative to squamous. A significantly (*p <*0.05) higher expression was also detected in female patients. COX-2 expression was not found to be correlated with either tumour stage or nodal status in our patient cohort, although a significant (*p *< 0.05) difference in expression was observed between grade 2 and grade 3 tumours. Correlation of COX-2 expression with 5-year patient survival revealed no significant correlation with overall survival, although a clear trend was observed for reduced survival in patients with high COX-2 expression (*p *= 0.2). However, COX-2 expression was significantly (*p <*0.01) associated with short-term (2-year) survival in our patient cohort, suggesting that this enzyme is prognostic in our study, at least for shorter-term survival (results shown in Table 1).

**Table 1:** Correlation of NSCLC TMA staining with clinical parameters

	COX-2 Staining Intensity (Mean ± SEM units)	*p*-Value	TXS Staining Intensity (Mean ± SEM units)	*p*-Value
**Gender**				
(n = 124) Male	28.9 ± 3.7	**0.03**	81.9 ± 3.4	**0.04**
(n = 73) Female	36.8 ± 4.1		98.6 ± 9.6	

**Tumour Subtype**				
(n = 83) Adenocarcinoma	39.5 ± 4.9	**0.02**	119 ± 7	**< 0.0001**
(n = 92) Squamous Cell Carcinoma	25.9 ± 3.7		58.9 ± 5.5	

**Tumour Stage**				
(n = 105) Stage 1	33.1 ± 3.5		93.3 ± 7.5	
(n = 43) Stage 2	35 ± 8.2	0.51	82.7 ± 9.3	0.7
(n = 43) Stage 3	26.9 ± 5		90 ± 9.7	

**Tumour Grade**				
(n = 13) Grade 1	27.4 ± 8.2	**0.02**	104.7 ± 21.7	**0.02**
(n = 106) Grade 2	38.9 ± 4.4	grade 2 *vs*.	98 ± 6.4	grade 2 *vs*.
(n = 64) Grade 3	26.9 ± 5	grade 3	70.4 ± 6.7	grade 3

**Nodal Status**				
(n = 119) Node Negative	35 ± 3.9	0.22	91.7 ± 6.3	0.4
(n = 79) Node Positive	27.6 ± 3.7		83.2 ± 7.1	

**2-Year Survival (months)**				
(n = 92) ≤ median value	21.8 ± 0.61	**0.008**	N/A	N/A
(n = 95) ≥ median value	18.8 ± 0.8			

**Overall Survival (months)**				
(n = 99) ≥ median value	47.4 ± 3.8		58.5 ± 3.6 months	
(n = 98) ≤ median value	54.2 ± 3.2	0.2	52.2 ± 3.7 months	0.32

**COX-2/TXS Combined Overall Survival (months)**				
(n = 22) High COX-2/Low TXS	N/A	N/A	45.5 ± 7.9 months	0.38
(n = 51) High COX-2/High TXS			54.1 ± 5 months	

Expression profile of COX-2 and TXS in a 204-patient NSCLC TMA: correlation with clinical parameters. Expression levels of COX-2 and TXS in NSCLC TMAs were correlated with a range of clinical parameters, including patient demographics, tumour classifications and overall survival.

NSCLC TMA analysis of thromboxane synthase expression revealed similar observations to those of COX-2 analysis. TXS expression levels were significantly (*p <*0.001) higher in adenocarcinoma patients, relative to squamous cell carcinoma. Expression was also significantly (*p <*0.05) higher in female patients. TXS expression did not correspond with either tumour stage or nodal status. However, expression levels were significantly (*p *< 0.05) lower in grade 3 tumours, relative to grade 2. No correlation of TXS expression with patient survival was observed. It has been established that TXS activity (and subsequent TXA_2 _generation) is dependent on COX-2 expression to supply the PGH2 substrate [22]. It may therefore be of importance to examine a potential role for COX-2 expression in combination with TXS expression in patient survival. Combined analysis revealed COX-2 expression to be significantly (*p *< 0.0001) correlated with that of TXS, with an r value of 0.34 (data not shown). However, no correlation of TXS expression in combination with high COX-2 with patient survival was observed, suggesting that thromboxane synthase is not a prognostic factor in NSCLC (results shown in Table 1).

### Thromboxane synthase expression in NSCLC cell lines: effects of selective inhibition on tumour cell growth

**Figure 3:**
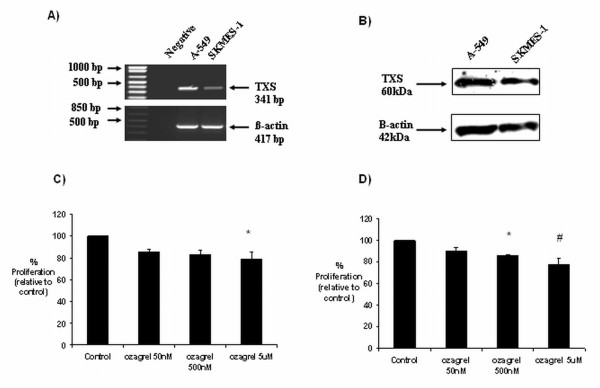
**Thromboxane synthase expression in NSCLC cells and effects of selective inhibition on tumour cell growth**. A modest basal expression of TXS was observed in A-549 and SKMES-1 cell lines at both the RNA (A) and protein (B) levels. Selective TXS inhibition resulted in a significant reduction in tumour cell survival in both A-549 (C) and SKMES-1 (D) cell lines. Data is expressed as mean ± SEM, with proliferation values expressed as a percentage of the controls (* *p <*0.05 relative to control, # *p <*0.05 relative to control; n = 3).

Two lung cancer cell lines-A-549 (adenocarcinoma) and SKMES-1 (squamous cell carcinoma)-were screened for TXS expression by both RT-PCR (Figure 3A) and western analysis (Figure 3B). A modest basal expression of both TXS mRNA and protein was observed in both cell lines, although mRNA expression in A-549 cells was higher than that in SKMES-1. The effect of 24 h selective thromboxane synthase inhibition with ozagrel on tumour cell proliferation/survival was then examined in both cell lines by BrdU cell proliferation assay. A significant (*p <*0.05) reduction in tumour cell proliferation/survival was observed in A-549 cells following treatment with 5 μM ozagrel, relative to untreated controls (79 ± 0.5% ozagrel *vs*. 100% untreated). A non-significant reduction in tumour cell proliferation/survival was also observed in these cells following treatment with 50 nM (85 ± 0.03%) and 500 nM (83 ± 0.04%) ozagrel, relative to controls (Figure 3C). A significant (*p <*0.05) reduction in tumour cell proliferation/survival was observed in SKMES-1 cells following 24 h treatment with 500 nM (86 ± 0.01%) and 5 μM (78 ± 0.02%) ozagrel, relative to untreated controls (Figure 3D).

### Tumour cell apoptosis is increased following selective thromboxane synthase inhibition

**Figure 4:**
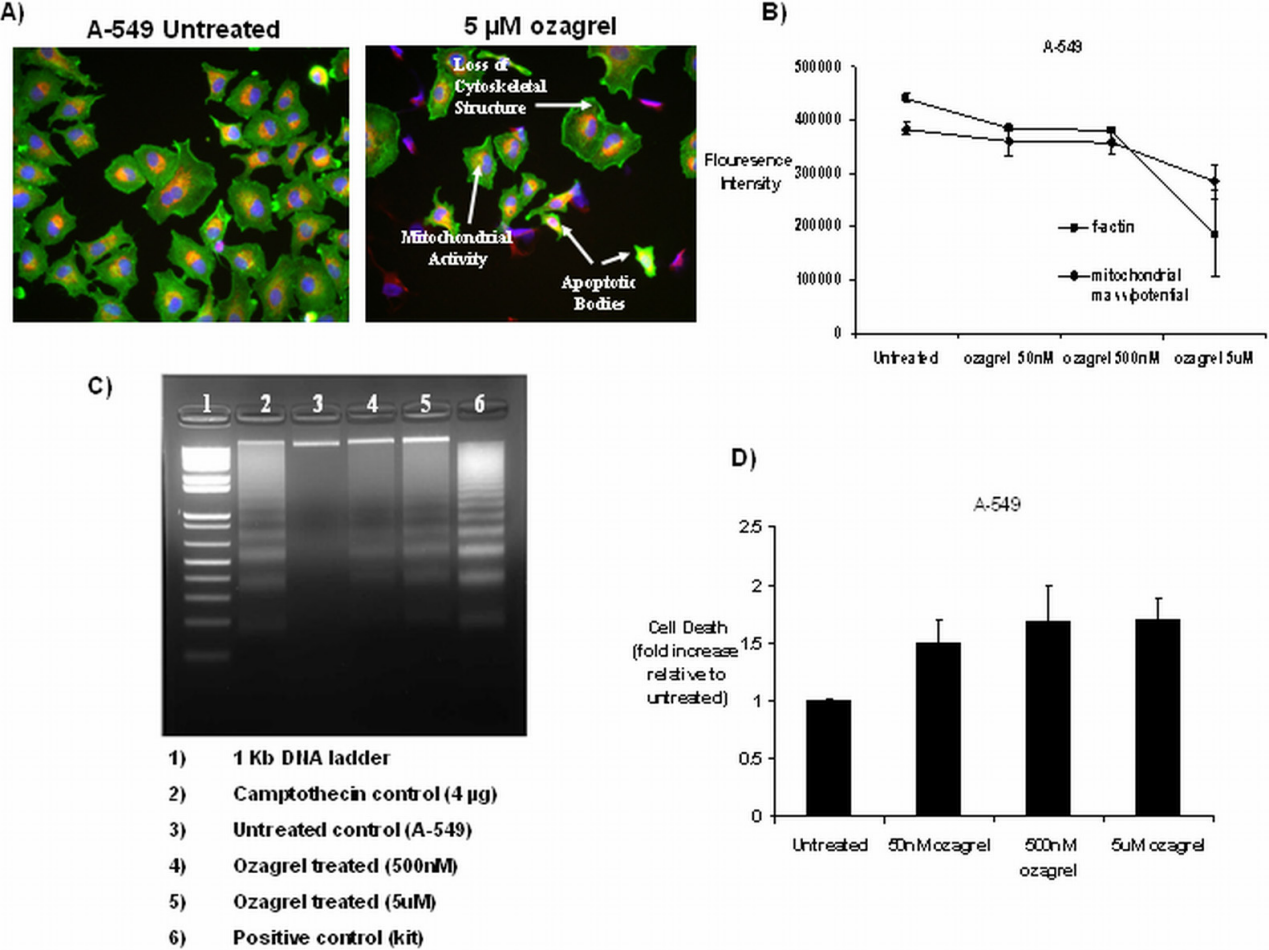
**The effects of TXS inhibition on tumour cell apoptosis**. A) Apoptosis was induced in a dose-dependant manner following 24 h treatment with ozagrel, relative to untreated control cells. Cell health following ozagrel treatment was assessed using 3 spectrally distinct flourophores to examine nuclei, *f*-actin (marker of cytoskeletal integrity), and mitochondrial mass/potential. Reduced *f*-actin levels demonstrate a loss in cellular integrity during apoptosis. Membrane blebbing also occurs and mitochondrial activity occurs, coupled with a loss of potential across the mitochondrial membrane. These markers were quantified by the Kinetic Scan HCS reader and are represented (B). Similar observations were made in SKMES-1 cells (data not shown). Apoptosis was confirmed following selective TXS inhibition by Cell Death Detection ELISA and DNA laddering in both cell lines (A-549 shown as representative). Cell Death ELISA demonstrated increased apoptosis in a concentration dependant manner, with fold induction expressed as a ratio of control cells (C). DNA laddering was also observed following ozagrel treatment at both 500 nM and 5 μM concentrations (D).

In order to further examine the effect of TXS inhibition on tumour cell survival pathways, High Content Screening Analysis of NSCLC cells was carried out following treatment. Multiparameter analysis of apoptosis signalling was assessed in both NSCLC cell lines following 24 h selective TXS inhibition using the GE In Cell Analyser. The Multiparamater Apoptosis HitKit from Cellomics provides High Content Screening qualified fluorescent reagents for simultaneous measurement of 3 fundamental parameters of apoptosis. Enlarged nuclei were stained blue (Hoechst), *f*-actin, a marker of cytoskeletal integrity, was stained green (Alexa Flour^® ^488 Phalloidin) and mitochondrial mass/potential within the cells was stained orange/pink (Mito Tracker^® ^Red). A notable change in the morphology of both cell lines was observed following treatment. These changes were characterised by a reduction in nuclear condensation and mitochondrial mass/potential within the cells, as well as a reduction in *f*-actin content (Figure 4A).

Quantification of multi-parameter apoptosis signalling was carried out using In Cell Analyser software. Selective TXS inhibition resulted in a reduction in both cellular *f*-actin content and mitochondrial mass/potential in both cell lines (Figure 4B), indicating that the mechanism whereby TXS inhibition reduces tumour cell proliferation/survival in these cells is through induction of apoptosis.

In order to confirm the observations of the High Content Screening, a number of alternate apoptosis assays were carried out on both cell lines following treatment with ozagrel. These included DNA laddering and Cell Death Detection ELISA. DNA fragmentation/laddering was observed in both cell lines following 24 h selective TXS inhibition (500 nM, 5 μM ozagrel; Figure 4C). In addition Cell Death Detection ELISA demonstrated increased nucleosome generation in the cytoplasmic fraction of cell lysates following TXS inhibition with 50 nM (1.5 ± 0.19 fold increase), 500 nM (1.68 ± 0.3 fold increase) or 5 μM (1.67 ± 0.18 fold increase) ozagrel in A-549 cells, relative to untreated controls (Figure 4D). A similar increase in nucleosome generation was observed in SKMES-1 cells following treatment with 50 nM (1.24 ± 0.03 fold increase), 500 nM (1.5 ± 0.13 fold increase) or 5 μM (1.65 ± 0.28 fold increase) ozagrel (data not shown). These results confirm that ozagrel exerts its effects through induction of apoptosis, resulting in DNA fragmentation, and nucleosome generation.

### Stable over-expression of thromboxane synthase in SKMES-1 cells

**Figure 5:**
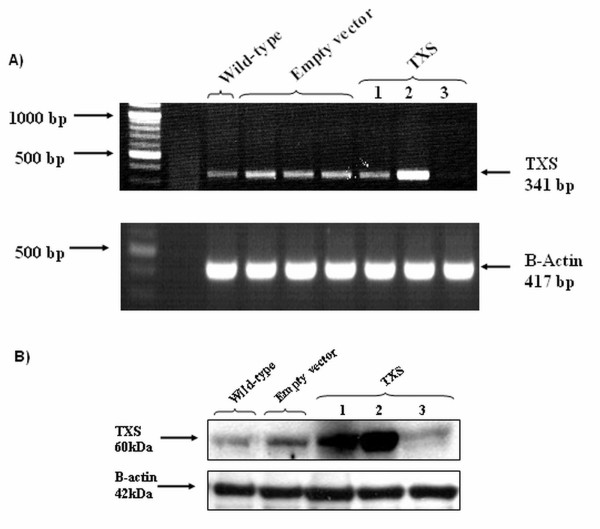
**Stable over-expression of TXS in SKMES-1 cells**. Following stable transfection, TXS over-expression was characterised by RT-PCR (A) and western analysis (B).

In order to confirm the effects of selective TXS inhibition, stable clones over-expressing TXS were generated in SKMES-1 cells. The SKMES-1 cell line was chosen for stable transfection of TXS as baseline expression of both RNA and protein levels of this enzyme in this cell line are relatively low (Figure 3A, B). Following transfection, TXS over-expression was characterised by both RT-PCR (Figure 5A) and western analysis (Figure 5B). A single TXS over-expressing clone was subsequently chosen for further analysis (*TXS *clone 2), while a corresponding empty vector clone was also selected as a control.

### Thromboxane synthase over-expression increases tumour cell growth/invasiveness and appears to protect against apoptosis

**Figure 6:**
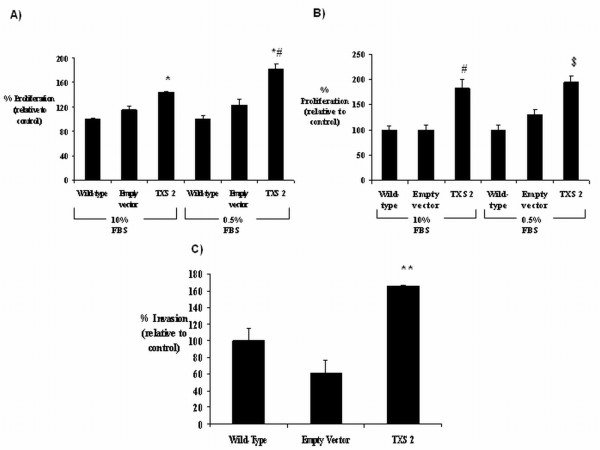
**Effect of stable *TXS*-over-expression on tumour cell growth and invasion**. Following characterisation of transfection efficiency, both *TXS *over-expressing and corresponding empty vector control clones were selected and cell proliferation/survival was examined by BrdU assay following 48 h (A) and 72 h (B) incubations. Data is represented at a percentage of the empty vector control, which was set to 100%. (* p < 0.05 *vs*. empty vector, *# p < 0.05 *vs*. 0.5% FBS empty vector, # p < 0.01 *vs*. empty vector, $ p < 0.01 *vs*. 0.5% FBS empty vector). Cell invasion was examined following 24 h incubation in stable transfected *TXS *clones (C). Each sample was loaded in triplicate onto the 96-well plate. Data is taken as a percentage of the empty vector control, which was set to 100%. (* p < 0.05 *vs*. empty vector, *# p < 0.05 *vs*. empty vector, ** p < 0.01 vs. empty vector). All data is expressed as mean ± SEM. Statistical analysis was carried out by ANOVA (one-way analysis of variance), with post-test analysis by Bonferroni multiple comparisons test. n = 3 independent experiments.

The effect of *TXS *over-expression on tumour cell proliferation/survival was examined by BrdU assay. Wild-type SKMES-1 cells, *TXS *over-expressing cells, and their corresponding empty vector control were seeded onto 96-well plates and allowed to adhere overnight. Cells were then either serum-starved (0.5% FBS media), or incubated in 10% FBS medium for 48 or 72 h. A significant increase (p < 0.05) in tumour cell growth was observed in *TXS *clone 2 when incubated under full-serum conditions for 48 h, relative to empty vector transfected controls (144 ± 2.6% *vs*. 115 ± 7.1%). A similar effect was observed when the cells were incubated in serum-depleted medium for 48 h (182 ± 8.3% clone 2 *vs*. 123 ± 9.4% empty vector). Empty vector transfected cells appeared to grow at a faster rate than wild-type cells under both full-serum (115 ± 7.1% *vs*. 100 ± 2.3%) and serum-depleted (123 ± 9.4% *vs*. 100 ± 5.5%) conditions, although this was not significant in either case (Figure 6A). A similar effect was observed in *TXS *over-expressing clone 2 following a 72 h incubation, with a highly significant increase (p < 0.01) in cell proliferation/survival observed under both full-serum (182.7 ± 17% clone 2 *vs*. 100.2 ± 8.6% empty vector) and serum-starved (194 ± 12.2% clone 2 *vs*. 130 ± 11.1% empty vector) conditions. While no difference in proliferation was observed between empty vector transfected cells and wild-type control cells under full-serum conditions (100.2 ± 8.6% *vs*. 100 ± 8.1%), proliferation was increased in empty vector cells following serum-starvation (130 ± 11.1% *vs*. 100 ± 10.6%), suggesting that these cells may be more robust under these conditions (Figure 6B).

TXS expression has been shown to have a potential role in tumour invasion and metastasis [26]. Invasion through the extra-cellular matrix (ECM) is an important step in tumour metastasis. The effect of TXS over-expression on tumour-cell invasion was therefore examined by Cell Invasion Assay. A significant increase in tumour-cell invasion was observed in *TXS*-transfected clone 2, relative to the corresponding empty vector control cells (165 ± 0.64% *vs*. 61.4 ± 15.4%). No significant difference in tumour cell invasion was observed between empty vector control SKMES-1 cells and wild-type cells (61.4 ± 15.4% vs. 100 ± 14.7%) (Figure 6C).

**Figure 7:**
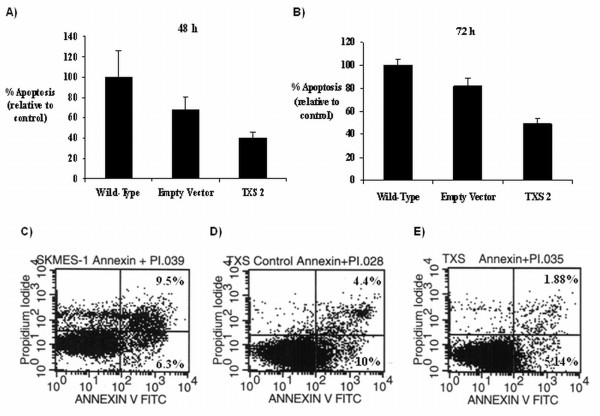
**Effect of stable *TXS*-over-expression on tumour cell survival**. Apoptosis was measured in *TXS *stable transfectants, and corresponding controls (wild-type and empty vector) following 48 h (A) and 72 h (B) serum-starvation by flow cytometry. Representative dot plots following 72 h serum starvation are shown for wild-type (C), empty vector (D) and TXS overexpressing (E) cells. Graphical data is represented at a percentage of the empty vector control, which was set to 100%. Data is expressed as mean ± SEM. n=3 independent experiments.

In order to further examine the effect of TXS over-expression on tumour survival pathways in SKMES-1 cells, tumour-cell apoptosis was examined in stable transfectant clones, corresponding empty vector controls, and wild-type controls by flow cytometry. Stable *TXS *over-expression appeared to protect against apoptosis in these cells. A reduction in tumour cell apoptosis was observed in the *TXS *clone 2 following 48 h (Figure 7A) or 72 h (Figure 7B) serum-starvation, relative to empty-vector transfected controls (40 ± 5% *vs*. 67 ± 12% 48 h, 49.6 ± 4.2% *vs*. 82.2 ± 6.6% 72 h). However, neither of these effects was found to be statistically significant. Additionally, no significant differences in apoptosis were observed between wild-type SKMES-1 cells and empty-vector control cells at either time-point. Representative dot plots showing percentage apoptosis in wild-type, empty vector, and TXS over-expressing clones following 72 h serum-starvation are shown in Figure 7C-E.

## Discussion

The tumour-promoting effects of COX-2 over-expression may be attributable to specific downstream products of arachidonic acid metabolism [30]. The function of thromboxane synthase and its prostanoid, thromboxane A_2 _in the cardiovascular system have been extensively studied and are well known. The enzyme has been shown to have a major role in maintaining cardiovascular haemostasis, mediated through its effects on platelet activation and aggregation. The impact of thromboxane synthase on cancer development and progression has received considerable interest over the past number of years. Expression of this enzyme has been shown to promote tumourigenesis *via *its effects on tumour growth, angiogenesis and metastasis [20-22,24]. However, the role of TXS in non-small cell lung cancer has not yet been investigated. The aim of the current study was to examine the expression profile of TXS in NSCLC and to determine whether expression of this enzyme is a prognostic factor or potential therapeutic target in the disease. Our study demonstrates that TXS expression is increased in NSCLC but was not a prognostic factor in the disease. However our data suggests that TXS promotes tumour growth and development, and is therefore a potential therapeutic target in NSCLC.

Examination of TXS expression pattern in matched tumour/normal protein samples revealed significantly increased expression in NSCLC samples relative to matched normal controls. TXS over-expression has been observed in a variety of other cancer types, including thyroid [20], bladder [21], prostate [22] and colorectal cancer [24]. TXS over-expression has been correlated with significantly reduced patient survival in bladder cancer [21]. In prostate cancer, its expression was significantly correlated with advanced stage and grade. In this study, the authors suggested that TXS activity was dependant on COX-2 and to a lesser degree, COX-1 to supply the PGH2 substrate [22,25]. Previous studies in human lung carcinoma found levels of the downstream metabolite of TXS, TXB_2_, to be significantly higher in tumour tissues relative to non-tumour controls [31]. Circulating TXS prostanoid levels cannot be measured quantitatively due to its very short half-life. In order to determine fractional amounts circulating in the plasma, the amount of the stable TXA_2 _metabolite, TXB_2 _was measured in a retrospective panel of NSCLC plasma samples and age-matched controls. TXB_2 _levels were significantly higher in patient samples, with basal control TXB_2 _levels comparing favourably to previous studies (unpublished data). Our observation suggests that circulatory TXB_2 _levels are increased in NSCLC, which may potentially impact on a patient's risk of developing venous thrombosis. Venous thromboembolism is a frequent cause of cancer-associated mortality [32] and patients with NSCLC have a 20-fold elevated risk of thrombosis [33]. In addition, increases in TXB_2 _expression have been associated with increased lipid peroxidation and bcl2 expression [31], suggesting that circulating TXB_2 _levels may promote tumour formation, partly *via *apoptosis evasion. As a result of our findings, we are setting up a study to prospectively examine the impact of circulating TXB_2 _levels on thromboembolic events and prognosis in NSCLC.

The role of COX-2 in lung cancer is well established, with a number of clinical trials examining COX-2 inhibition as a potential therapy for NSCLC [9-11]. COX-2 expression is required for PGH2 and subsequent prostanoid generation (including thromboxane A_2_), and is therefore of importance for the activity and subsequent effects of TXS. This is supported by a previous report that TXS regulates tumour motility *via *the COX-2 pathway in prostate cancer [22]. Thromboxane A_2_, the prostanoid product of TXS, has also been shown to mediate COX-2 dependant angiogenesis. Linear correlation analysis revealed a highly significant association of COX-2 with TXS expression, supporting previous observations [22]. COX-2 and TXS expression levels were both significantly higher in adenocarcinoma patients, relative to squamous. The significance of these observations is unclear, but it has been observed that patients with adenocarcinoma are at a higher risk of developing venous thrombosis than those with squamous cell carcinoma [33]. A significant increase in COX-2 and TXS expression was also observed in female patients, relative to males. While no studies have previously been carried out to determine a possible gender influence on the expression of these enzymes in cancer, recent studies in murine models have shown that levels of constrictor prostanoids such as COX-2 and TXA_2 _are higher in females than males [34]. Expression levels of both enzymes were not altered with either tumour stage or nodal status. Interestingly, both COX-2 and TXS expression levels were significantly lower in grade 3 tumours, relative to grade 2. These observations are in contrast to those in a prostate cancer study [22], but are supported by previous observations in breast cancer [35], suggesting that the expression pattern of this enzyme may vary between cancer types.

COX-2 expression has previously been associated with reduced patient survival in NSCLC [6-8]. Our study noted similar observations. While COX-2 expression was not associated with a significant reduction in long-term (5-year) survival, statistical significance was observed for short-term (2-year) patient survival. In contrast, no significant correlation of TXS expression with patient survival was observed, even when TXS expression was examined in combination with levels of COX-2 expression. While this observation may seem surprising, given previous findings, similar observations have been observed in breast cancer patients where low levels of TXS expression were correlated with tumours of high grade and a predicted poor clinical outcome [35]. The authors also found that levels of the thromboxane receptor (TP) were negatively associated with disease-free survival, suggesting that the downstream receptor, as opposed to its corresponding synthase, may be prognostic in the disease [36].

Selective inhibition of TXS has been shown to reduce tumour cell proliferation/survival in a number of cancers including colorectal carcinoma [24], and bladder cancer [21]. In our study, selective inhibition of TXS significantly (and dose-dependently) reduced tumour cell growth in an adenocarcinoma and squamous cell NSCLC cell line. This was mirrored by a reduction in thromboxane metabolite (TXB_2_) generation, as assessed by enzyme immunoassay of the culture medium (data not shown). In direct contrast, stable TXS over-expression significantly increased tumour cell growth in SKMES-1 cells, implicating this enzyme as a survival factor and potential therapeutic target in NSCLC. Our observations are in agreement with previous observations in a xenograft colorectal cancer model [27], and suggest that TXS is a potential therapeutic target in NSCLC pathogenesis. While the effect of TXS inhibition on healthy cells was not examined, previous studies have shown that TXS blockade inhibits endothelial cell migration in response to VEGF or bFGF, suggesting an effect on normal cellular mechanisms [23]. The significantly increased NSCLC TXS levels observed in our study suggests that TXS inhibition would have a preferential effect on tumour cells and warrants further investigation in NSCLC pathogenesis.

Increased apoptosis following TXS inhibition was demonstrated by High Content Screening (HCS), and quantified using In Cell Analyser software. Apoptosis was characterised by a reduction in mitochondrial mass/potential and *f*-actin content in both cell lines following treatment with ozagrel. HCS observations were validated by DNA laddering and Cell Death Detection ELISA. These findings are consistent with a recent study by Moussa *et al. *in bladder cancer [26]. They are also supported by our further observations that stable TXS over-expression appeared to protect against tumour cell apoptosis, relative to controls. It may therefore be hypothesised that TXS exerts its effects on tumour cells, at least in part, *via *inhibition of apoptosis. In addition to apoptosis however, TXS and its product, TXA_2 _have also been implicated in the regulation of other cancer survival pathways such as angiogenesis [23], migration [21,22], invasion [21], and tumour cell metastasis [23]. In our study TXS over-expression significantly increased the invasive capacity of SKMES-1 cells, suggesting another survival mechanism associated with over-expression. In order to further examine the mechanism underlying the effects of selective TXS inhibition, RT^2 ^Profiler™ Cancer PathwayFinder PCR arrays were used to examine the expression of a panel of genes implicated in tumourigenesis following 24 h treatment with ozagrel (additional file 1). Notable changes in gene expression following treatment were observed in pathways of apoptosis, angiogenesis and invasion/metastasis. While each cell line exhibited distinct gene expression profiles following TXS inhibition, a small subset of genes stood out as being of interest. These included the pro-apoptotic genes *Bax *(4-fold increase), *Granzyme A *(9-fold increase), and *TNF *(4-fold increase) in A-549 cells and anti-apoptotic genes *Bcl2 *(3-fold decrease) and *IGF1 *(5-fold decrease) in SKMES-1 cells (Additional file 2, Figure S1). The previous observation that increases in circulating TXB_2 _expression is associated with BCL2 expression lends support to our observations [31]. In addition to the effects of TXS inhibition on tumour cell survival mechanisms, examination of downstream genetic pathways implicated in tumourigenesis is also of particular interest in NSCLC, with the potential to provide further insight into the anti-tumour effects of TXS inhibition.

In conclusion, TXS and its metabolite, TXB_2_, are over-expressed in NSCLC. While TXS was not found to be prognostic, our study clearly shows that it is pro-tumourigenic and may be a target for therapeutic intervention *via *induction of apoptosis. Inhibition of this enzyme may therefore be a suitable therapeutic approach for NSCLC treatment, either alone, or in combination with conventional chemotherapeutic agents.

## Abbreviations

NSCLC: non-small cell lung cancer; AA: arachidonic acid; COX: cyclooxygenase; TXS: thromboxane synthase; TXA_2_: thromboxane A_2_; TXB_2_: thromboxane B_2_; TMA: tissue microarray; IHC: immunohistochemistry.

## Competing interests

The authors declare that they have no competing interests.

## Authors' contributions

MCC carried out all *in-vitro *experiments (with the exception of high content screening) and drafted the manuscript. KG carried out high content screening analysis. KG also generated the NSCLC tissue microarrays, carried out TMA staining analysis (TXS and COX-2) and collected/isolated NSCLC protein and plasma samples (and relevant controls). RC aided in the generation of the NSCLC tissue microarrays. R.C. also cut sections from these TMAs and assisted in optimisation of both TXS and COX-2 IHC staining. EK participated in the design of the study and also provided technical (pathological) expertise in relation to the staining and grading of NSCLC tissue microarrays (and full-face sections) for both TXS and COX-2. KJO'B participated in the design and coordination of the study and provided technical expertise when drafting the manuscript.  GPP played a major role in the design and coordination of the study. GPP also carried out TMA staining analysis (TXS and COX-2) and helped draft the manuscript. All authors read and approved the final manuscript.

## Supplementary Material

Additional file 1 **Examination of changes in cancer-associated gene expression profile following selective TXS inhibition**. Changes in gene expression profile following selective TXS inhibition were assessed in both A-549 and SKMES-1 cell lines by quantitative PCR array, using RT^2 ^Profiler™ Cancer PathwayFinder arrays. Click here for file

Additional file 2 **Pie charts demonstrating the changes in cancer-associated gene expression profile following selective TXS inhibition (in A-549 and SKMES-1 NSCLC cell lines)**. The results of the analysis described in additional file 1 are represented graphically in this additional file (Additional File 2, Figure S1), with pie charts used to group qRT-PCR changes in gene expression following TXS inhibition into the 6 hallmarks of cancer. Figure S1: Cancer-gene expression profiling following 24 h selective TXS inhibition in A-549 (A) and SKMES-1 (B) cell lines. Genes were grouped according to the six hallmarks of cancer. These included genes involved in cell cycle and DNA damage repair, apoptosis/cell senescence, signal transduction/transcription, adhesion, angiogenesis and invasion/metastasis. Click here for file
