# Supplementary material for: Examination of thromboxane synthase as a prognostic factor and therapeutic target in non-small cell lung cancer
Source: Mol Cancer. 2011 Mar 9;10:25. doi: 10.1186/1476-4598-10-25 (PMC3074522; doi:10.1186/1476-4598-10-25)
Supplement: Additional file 1 — Examination of changes in cancer-associated gene expression profile following selective TXS inhibition. Changes in gene expression profile following selective TXS inhibition were assessed in both A-549 and SKMES-1 cell lines by quantitative PCR array, using RT2 Profiler™ Cancer PathwayFinder arrays. [file 1476-4598-10-25-S1.DOCX]

**Additional Data:**

**Methods:**

**Quantitative PCR Analysis**

**Cancer Pathway Finder Arrays**

Changes in gene expression profile following selective TXS inhibition were assessed in both A-549 and SKMES-1 cell lines by quantitative PCR array, using RT² Profiler™ Human Cancer PathwayFinder PCR Arrays (SuperArray Bioscience Corporation, MD, USA). Briefly, cells were grown in 75cm^2^ flasks, incubated overnight in serum-depleted medium, and then treated with 500 nM ozagrel for 24 h. Total RNA was isolated using Tri-Reagent (MRC, OH, USA) and prepared using the Qiagen RNeasy® Mini Kit according to manufacturers’ instructions, including DNAse treatment (Qiagen Ltd., Sussex, UK). First strand cDNA was synthesized using the ReactionReady^TM^ First Strand cDNA Synthesis kit (Molecular Research Center, Montgomery Road, OH, USA). A RT-PCR reaction mixture was made consisting of 98 μL of the cDNA product and 1225 μL RT² Real-Time™ SYBR Green PCR Master-Mix in 2450 μL of PCR reaction, of which 25 μL was added to each well of the PCR array. qPCR reactions were performed using ABI 7500 instrumentation for 40 cycles with denaturation at 95°C for 15 s and an annealing at 60°C for 1 min. The threshold cycle for each well was calculated using the instruments software, and the fold-change for each gene was calculated using SuperArray's PCR array data analysis template.

**Results:**

**Changes in gene expression profile following selective thromboxane synthase inhibition: cancer-pathway profiler arrays**

In order to examine the potential gene-mechanisms underlying the effects of TXS inhibition, quantitative PCR was carried out using Human Cancer Pathway Finder PCR Arrays (SuperArray Bioscience Corporation, MD, USA) following 24 h treatment with 500 nM ozagrel. A panel of genes implicated in tumourigenesis were found to be differentially regulated in both A-549 and SKMES-1 cell lines (greater than 2-fold change) following TXS inhibition, relative to untreated controls. A total of 7 genes were altered (up-regulated or down-regulated) in A-549 cells, with 11 genes altered in SKMES-1 cells. Changes in gene-expression following selective TXS inhibition are represented graphically in the form of pie-charts, with genes grouped according to the six main hallmarks of cancer (Additional file 2, Figure S1).

The most common changes in gene-expression following selective TXS inhibition were observed in the apoptosis/cell senescence, angiogenesis, and invasion/metastasis biological pathways. Within the apoptosis group up-regulation in the expression of pro-apoptotic genes *Bax* (4.3 fold) and *Granzyme A* (8.7 fold) was observed in A-549 cells, while the anti-apoptotic gene *Bcl2* was down-regulated (3-fold) in SKMES-1 cells. Angiogenesis genes which were differentially altered included *IGF1* (3-fold increase) and *TNF* (4-fold increase) in A-549 cells and *IGF1* (5-fold decrease), *TNF* (4-fold decrease), *ANGPT1* (2-fold increase), *ANGPT2* (3-fold increase) *TEK* (4-fold decrease) *IFNA1* (2-fold decrease) and *IGNB1* (2-fold decrease) in SKMES-1 cells. Finally alterations in genes involved in invasion/metastasis following TXS inhibition included *MMP1* (4-fold decrease) and *MMP9* (7-fold increase) in A-549 cells and *MMP1* (3-fold increase) and *TIMP3* (4-fold decrease) in SKMES-1 cells.
