# Supplementary figures and images for: Examination of thromboxane synthase as a prognostic factor and therapeutic target in non-small cell lung cancer
Source: Mol Cancer. 2011 Mar 9;10:25. doi: 10.1186/1476-4598-10-25 (PMC3074522; doi:10.1186/1476-4598-10-25)

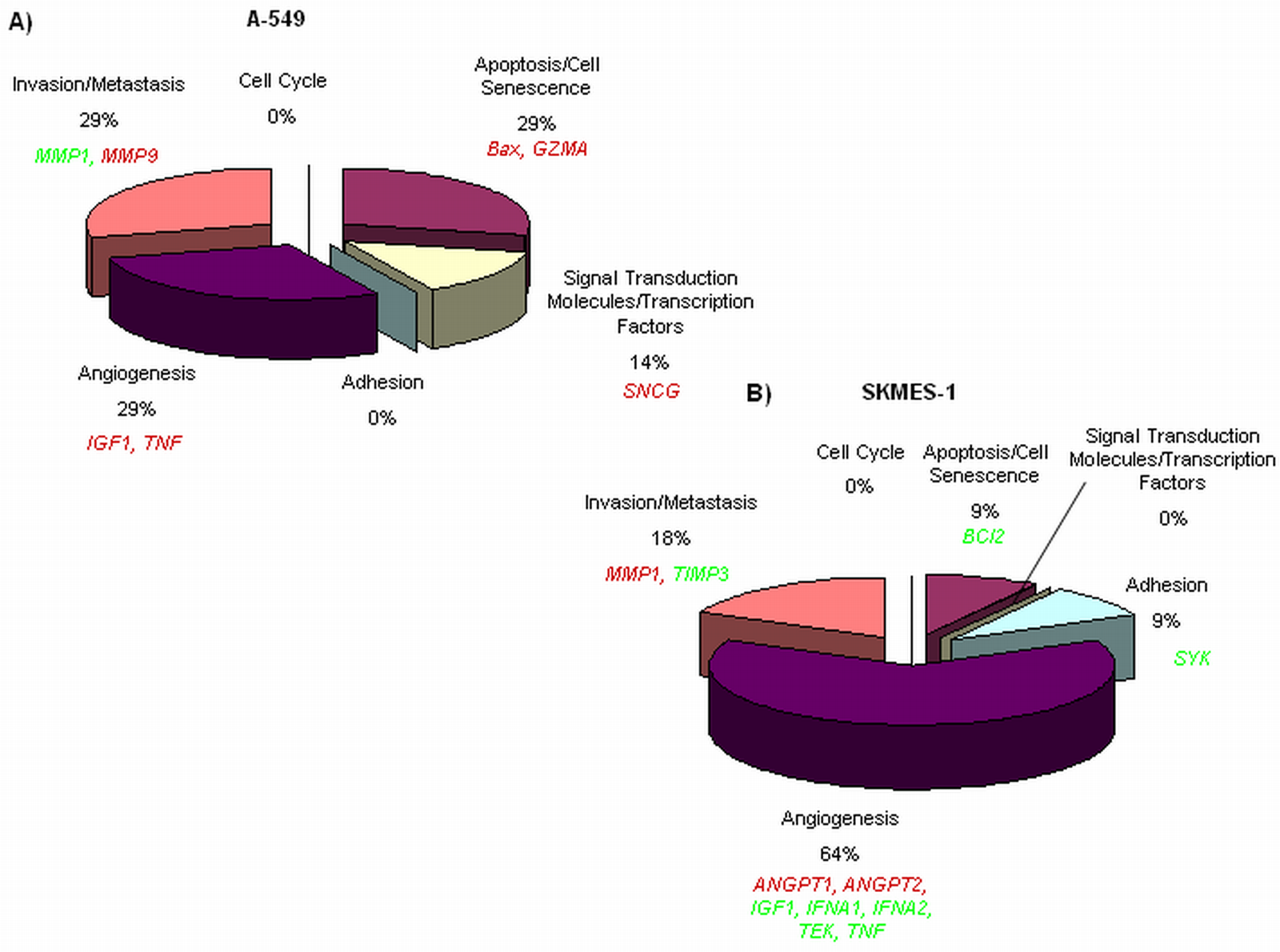

Supplement: Additional file 2 — Pie charts demonstrating the changes in cancer-associated gene expression profile following selective TXS inhibition (in A-549 and SKMES-1 NSCLC cell lines). The results of the analysis described in additional file 1 are represented graphically in this additional file (Additional File 2, Figure S1), with pie charts used to group qRT-PCR changes in gene expression following TXS inhibition into the 6 hallmarks of cancer. Figure S1: Cancer-gene expression profiling following 24 h selective TXS inhibition in A-549 (A) and SKMES-1 (B) cell lines. Genes were grouped according to the six hallmarks of cancer. These included genes involved in cell cycle and DNA damage repair, apoptosis/cell senescence, signal transduction/transcription, adhesion, angiogenesis and invasion/metastasis. [file 1476-4598-10-25-S2.TIFF]
